# Ecologic analysis of antimicrobial use in South Carolina hospitals during 2020–2022

**DOI:** 10.1017/ash.2023.496

**Published:** 2023-12-12

**Authors:** Pamela Bailey, Shujie Chen, Majdi N. Al-Hasan, Bankole Olatosi, Xiaoming Li, Jiajia Zhang

**Affiliations:** 1 Department of Internal Medicine, Division of Infectious Diseases, Prisma Health—Midlands, Columbia, SC, USA; 2 University of South Carolina School of Medicine, Columbia, SC, USA; 3 Department of Epidemiology and Biostatistics, Arnold School of Public Health, University of South Carolina, Columbia, SC, USA; 4 Big Data Health Science Center, Arnold School of Public Health, University of South Carolina, Columbia, SC, USA; 5 Department of Health Services Policy and Management, Arnold School of Public Health, University of South Carolina, Columbia, SC, USA; 6 Department of Health Promotion, Education, and Behavior, Arnold School of Public Health, University of South Carolina; Columbia, SC, USA

## Abstract

**Background:**

Factors influencing excessive antimicrobial utilization in hospitalized patients remain poorly understood, particularly with the COVID-19 pandemic.

**Methods:**

In this retrospective cohort, we compared administrative data regarding antimicrobial prescriptions in hospitalized patients in South Carolina from March 2020 through September 2022. The study examined variables associated with antimicrobial use across demographics, COVID status, and length of stay, among other variables.

**Results:**

Significant relationships were seen with antimicrobial use in COVID-19 positive patients (OR 2.00, 95% Confidence Interval (CI): 1.9–2.1), young adults (OR 1.08, 95% CI: 0.99–1.12, COVID-19 positive Blacks and Hispanics (OR 1.06, 95% CI: 1.01–1.11, OR 1.05, 95% CI: 0.89–1.23), and COVID-19 positive patients with ≥2 comorbid conditions (OR 1.55, 95% CI: 1.43–1.68).

**Discussion:**

Further analysis in more than one healthcare system should explore these ecologic relationships further to understand if these are common trends to inform ongoing stewardship interventions.

## Introduction

Approximately 50% of hospitalized patients in the United States (US) receive antibiotics, with little change from this baseline in the last ten years, despite the US *National Action Plan to Combat Antibiotic Resistant Bacteria* goal to reduce inappropriate antibiotic use by 20% in inpatient setting.^1,2^ The intersection of the COVID-19 pandemic and this ongoing urgent public health threat of antimicrobial resistance created antimicrobial stewardship (ASP) challenges juxtaposed with the lack of knowledge about the disease state of COVID-19. Antibiotic prescribing in early COVID-19 patients in Europe was 63.1%; in the USA, 64.8%; in China, 76.2%; in the Middle East, 86.0%; and in East/Southeast Asia (excluding China), 87.5%.^3^ There was a trend toward reduced antibiotic prescribing as the pandemic continued, as studies ending in April 2020 showing 62.6%, down from 85.3%^3^; this remains higher than the baseline pre-pandemic and is clearly higher than the goal of reduced antimicrobial use by the US action plan.

A Canadian interrupted time series analysis from March 2019 to June 2021 through three pandemic waves showed only increased antimicrobial use in the first wave of the pandemic in the general wards, but above baseline antimicrobial use in waves 1 and 2 of the pandemic in the ICU.^4^ In an evaluation of twelve hospitals from January 2019 to February 2021 with different ASP models, there were some fluctuations in antimicrobial prescribing practices, however, no statistically significant deviations from previous trends.^5^ In a study comparing antimicrobial use in hospitals that admitted vs those that did not admit COVID-19 patients in the early pandemic, a 6.6% increase in antimicrobial use was seen in those that did admit COVID-19 patients. Most of the antimicrobial use seen was in broad-spectrum antimicrobials, including a 16.4% increase in broad-spectrum agents primarily used to treat hospital-onset infections and a 9.9% increase in the use of anti-methicillin-resistant *Staphylococcus aureus* agents.^6^

There is a significant amount of inappropriate antimicrobial utilization regardless of COVID-19, and it is poorly understood who is receiving these antimicrobials. In this analysis, we compared administrative data regarding antimicrobial prescriptions in hospitalized patients, including an analysis of those with COVID-19 diagnoses to those who did not have COVID-19. We attempted to characterize patient populations that received excessive antibiotics in South Carolina (SC) hospitals to attempt to better understand overall population-based antimicrobial use and target appropriate ASP interventions.

## Methods

### Population and data source

The study population includes patients who had inpatient clinical visit encounters at a major healthcare system in SC between March 2020 and September 2022. This system services 2,700 beds in multiple hospitals and affiliates, and their electronic medical record system was the source of an administrative dataset from these deidentified patient encounters. The database provided information regarding visit information (admission date, admission source, encounter type, primary payor, discharge date, and discharge type), disease diagnosis code (International Classification of Diseases 10^th^ revision [ICD-10] code), and medication prescribing information (prescribing order/start/end date, dosage, and frequency). The data were integrated by the SC Office of Revenue and Fiscal Affairs (RFA), and only deidentified data were released to the research team for analysis. The dates included in the dataset were offset (1–365) to further deidentify the data. After excluding patients for missing data (72 with erroneous linkage ID, 65 patients with erroneous age records, 5,304 with age <18 years old, 1 patient with missing gender, and 6 with missing residence), 33,943 patients were included with overall 63,916 inpatient encounters (4,010 COVID-19 positives and 59,906 negatives) as the study population for the overall cohort. We further define two sub-cohorts: patients with diabetes mellitus, type II (DM2) (*N* = 10,723 with 24,551 inpatient encounters) and patients with obesity (*N* = 8,453 with 18,327 inpatient encounters) as part of the diagnoses for subgroup analysis.

## Definitions

The RxNorm code was used to identify the antimicrobial medications; the RxNorm is standard clinical drug vocabulary used by the National Library of Medicine to capture all drugs regardless of manufacturer.^7^ We defined the outcome antimicrobial use as a binary variable, indicating whether the patient has any antimicrobial ordered during an inpatient visit type (see Supplemental Data).

Demographics include age group, gender, race (White, Black, other, unknown/missing), ethnicity (Hispanic/Latino, non-Hispanic/Latino, and unknown/missing), and residence (rural, urban). Rural residences are counties that are not designated as part of Metropolitan Areas by the Office of Management and Budget.^8^ Note that this is limited to what the electronic medical record (EMR) captures, including binary genders, race, and ethnicity classifications as is pre-set for capture at intake.

The COVID-19 and comorbid condition diagnoses were identified based on the ICD-10 code. We defined 19 comorbidities categories (see Table S10) utilizing Centers for Disease Control and Prevention definitions for patients with certain medical conditions at high risk for COVID-19 complications.^9^ When adjusting for comorbidities in the model, we categorized it into three levels based on number of comorbid conditions: 0, 1, ≥2. For subanalyses, we categorized the number of comorbidities besides DM2 or obesity into three levels: 1, 2, ≥3 since all of them had at least one comorbidity besides DM2 or obesity. Length of stay was defined as days from admission date to discharge date and was categorized into five levels: 0–2, 3–6, 7–13, 14–28, ≥29 days.

### Statistical analysis

Descriptive statistics were used to summarize the distribution of antimicrobial use across characteristics for overall, COVID-19 diagnosis, and COVID-19 negative cohorts, respectively. The demographic subgroups were compared via chi-square test or Fisher’s exact test as appropriate. Generalized linear mixed models (GLMMs) with logistic link and autoregression covariance matrix (selected based on Quasi information criterion) were applied to investigate the potential risk factors for antimicrobial use. We examine the impact of COVID-19 status, demographic, comorbidity, and length of stay step by step. Model 1 adjusted for COVID-19 status only, while model 2 overlaid demographics, and then model 3.a and model 3.b overlaid number of comorbidities and length of stay, respectively. Subsequently, model 4.a and model 4.b evaluated possible interactions between COVID-19 status and other covariates and we only reported the model with the significant interaction. Similar GLMM models were applied to the DM2 and obesity subgroups separately. The odds ratio (OR) and 95% confidence interval (CI) were reported. *P*-values under 0.05 were considered statistically significant. All statistical analysis was conducted using statistical software, SAS version 9.4 (SAS Institute, Inc., Cary, NC, USA) and R 4.2.0 (R Core Team 2022).

## Results

### Overall

**Figure 1.**
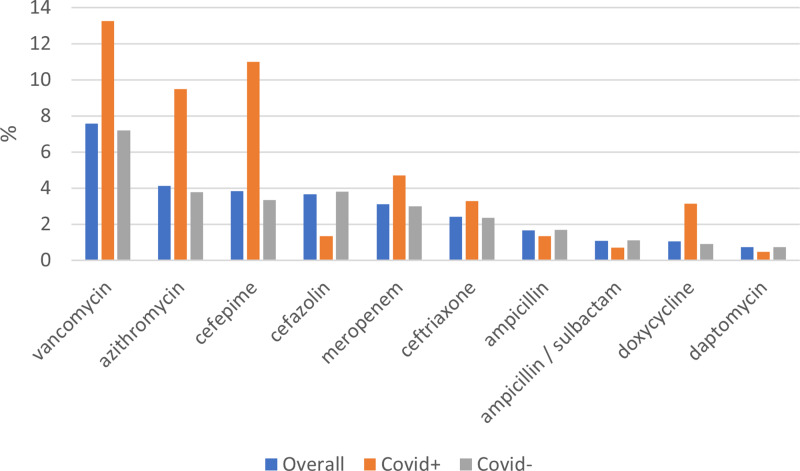
Top 10 frequencies of medication prescription by COVID-19 ICD-10 code.

Of 63,916 inpatient encounters in the overall population, 14,190 (22.2%) had antimicrobial use during the 2020–2022 period of analysis (Table 1). There was a significant difference in antimicrobial use in those who were COVID-19 diagnosed compared to those without COVID-19 diagnosis (34.9% vs 21.4%, *p* < 0.0001) (Tables 2 and 3). Vancomycin, azithromycin, and cefepime were the three most commonly used antibiotics in hospitalized patients with COVID-19 diagnosis in the respective order (Figure 1 and Table 2). Patients with antimicrobial use were younger, more likely to be Black and of Hispanic or Latino ethnicity (Table 1). Patients with ≥2 comorbidities and residents in urban counties were also more likely to receive antimicrobials than those with <2 comorbidities and residents of rural counties, respectively (Table 1).

**Table 1.** Demographic characteristics of the overall inpatient encounters

Characteristics	Overall	No antibiotics	Any antibiotics	*p*-value
	*N* = 63,916	*N* = 49,726	*N* = 14,190	
		77.80%	22.20%	
Age group				<.0001
18–29	6,633 (10.38)	5,033 (10.12)	1,600 (11.28)	
30–39	7,695 (12.04)	5,739 (11.54)	1,956 (13.78)	
40–49	7,146 (11.18)	5,647 (11.36)	1,499 (10.56)	
m50–59	10,330 (16.16)	8,271 (16.63)	2,059 (14.51)	
≥60	32,112 (50.24)	25,036 (50.35)	7,076 (49.87)	
Gender				0.999
Female	34,890 (54.59)	27,144 (54.59)	7,746 (54.59)	
Male	29,026 (45.41)	22,582 (45.41)	6,444 (45.41)	
Race				<.0001
White	35,202 (55.08)	27,596 (55.50)	7,606 (53.60)	
Black	27,655 (43.27)	21,345 (42.93)	6,310 (44.47)	
Other	337 (0.53)	254 (0.51)	83 (0.58)	
Unknown/Missing	722 (1.13)	531 (1.07)	191 (1.35)	
Ethnicity				0.0171
Not Hispanic or Latino	62,180 (97.28)	48,412 (97.36)	13,768 (97.03)	
Hispanic or Latino	1,421 (2.22)	1,063 (2.14)	358 (2.52)	
Unknown/Missing	315 (0.49)	251 (0.50)	64 (0.45)	
Residence				<.0001
Rural	12,852 (20.11)	10,377 (20.87)	2,475 (17.44)	
Urban	51,064 (79.89)	39,349 (79.13)	11,715 (82.56)	
Covid-19 status				<.0001
Negative	59,906 (93.73)	47,115 (94.75)	12,791 (90.14)	
Positive	4,010 (6.27)	2,611 (5.25)	1,399 (9.86)	
# of comorbidities				<.0001
0	5,394 (8.44)	4,373 (8.79)	1,021 (7.20)	
1	4,651 (7.28)	3,868 (7.78)	783 (5.52)	
≥2	53,871 (84.28)	41,485 (83.43)	12,386 (87.29)	
Length of stay (days)				<.0001
0–2	18,971 (29.68)	16,709 (33.60)	2,262 (15.94)	
3–6	26,470 (41.41)	20,896 (42.02)	5,574 (39.28)	
7–13	12,449 (19.48)	8,832 (17.76)	3,617 (25.49)	
14–28	4,729 (7.40)	2,770 (5.57)	1,959 (13.81)	
29+	1,297 (2.03)	519 (1.04)	778 (5.48)	

**Table 2.** Demographic characteristics of the inpatient COVID-19 diagnosis during hospital encounters

Characteristics	Overall	No med	Any med	*p*-value
	*N* = 4,010	*N* = 2,611	1,399	
		65.11%	34.89%	
Age group				0.4021
18–29	253 (6.31)	175 (6.70)	78 (5.58)	
30–39	312 (7.78)	206 (7.89)	106 (7.58)	
40–49	379 (9.45)	252 (9.65)	127 (9.08)	
50–59	571 (14.24)	380 (14.55)	191 (13.65)	
≥60	2,495 (62.22)	1,598 (61.20)	897 (64.12)	
Gender				0.001
Female	2,217 (55.29)	1,493 (57.18)	724 (51.75)	
Male	1,793 (44.71)	1,118 (42.82)	675 (48.25)	
Race				0.0018
White	1,697 (42.32)	1,159 (44.39)	538 (38.46)	
Black	2,194 (54.71)	1,383 (52.97)	811 (57.97)	
Other	18 (0.45)	*	*	
Unknown/Missing	101 (2.52)	*	*	
Ethnicity				0.7114
Not Hispanic or Latino	3,831 (95.54)	2,499 (95.71)	1,332 (95.21)	
Hispanic or Latino	161 (4.01)	*	*	
Unknown/Missing	18 (0.45)	*	*	
Residence				0.0001
Rural	990 (24.69)	695 (26.62)	295 (21.09)	
Urban	3,020 (75.31)	1,916 (73.38)	1,104 (78.91)	
# of comorbidities				<.0001
0	262 (6.53)	200 (7.66)	62 (4.43)	
1	303 (7.56)	227 (8.69)	76 (5.43)	
≥2	3,445 (85.91)	2,184 (83.65)	1,261 (90.14)	
Length of stay (days)				<.0001
0–2	666 (16.61)	520 (19.92)	146 (10.44)	
3–6	1,573 (39.23)	1,188 (45.50)	385 (27.52)	
7–13	1,077 (26.86)	658 (25.20)	419 (29.95)	
14–28	520 (12.97)	206 (7.89)	314 (22.44)	
29+	174 (4.34)	39 (1.49)	135 (9.65)	

Note.

* Small number of less than 10 were masked due to SC DHEC’s policy.

**Table 3.** Demographic characteristics of the inpatient COVID-19 negative encounters

Characteristics	Overall	No med	Any med	*p*-value
	*N* = 59,906	*N* = 47,115	*N* = 12,791	
		78.65%	21.35%	
Age group				<.0001
18–29	6,380 (10.65)	4,858 (10.31)	1,522 (11.90)	
30–39	7,383 (12.32)	5,533 (11.74)	1,850 (14.46)	
40–49	6,767 (11.30)	5,395 (11.45)	1,372 (10.73)	
50–59	9,759 (16.29)	7,891 (16.75)	1,868 (14.60)	
≥60	29,617 (49.44)	23,438 (49.75)	6,179 (48.31)	
Gender				0.3598
Female	32,673 (54.54)	25,651 (54.44)	7,022 (54.90)	
Male	27,233 (45.46)	21,464 (45.56)	5,769 (45.10)	
Race				0.1227
White	33,505 (55.93)	26,437 (56.11)	7,068 (55.26)	
Black	25,461 (42.50)	19,962 (42.37)	5,499 (42.99)	
Other	319 (0.53)	245 (0.52)	74 (0.58)	
Unknown/Missing	621 (1.04)	471 (1.00)	150 (1.17)	
Ethnicity				0.1144
Not Hispanic or Latino	58,349 (97.40)	45,913 (97.45)	12,436 (97.22)	
Hispanic or Latino	1,260 (2.10)	963 (2.04)	297 (2.32)	
Unknown/Missing	297 (0.50)	239 (0.51)	58 (0.45)	
Residence				<.0001
Rural	11,862 (19.80)	9,682 (20.55)	2,180 (17.04)	
Urban	48,044 (80.20)	37,433 (79.45)	10,611 (82.96)	
# of comorbidities				<.0001
0	5,132 (8.57)	4,173 (8.86)	959 (7.50)	
1	4,348 (7.26)	3,641 (7.73)	707 (5.53)	
≥2	50,426 (84.18)	39,301 (83.42)	11,125 (86.98)	
Length of stay (days)				<.0001
0–2	18,305 (30.56)	16,189 (34.36)	2,116 (16.54)	
3–6	24,897 (41.56)	19,708 (41.83)	5,189 (40.57)	
7–13	11,372 (18.98)	8,174 (17.35)	3,198 (25.00)	
14–28	4,209 (7.03)	2,564 (5.44)	1,645 (12.86)	
29+	1,123 (1.87)	480 (1.02)	643 (5.03)	

**Figure 2.**
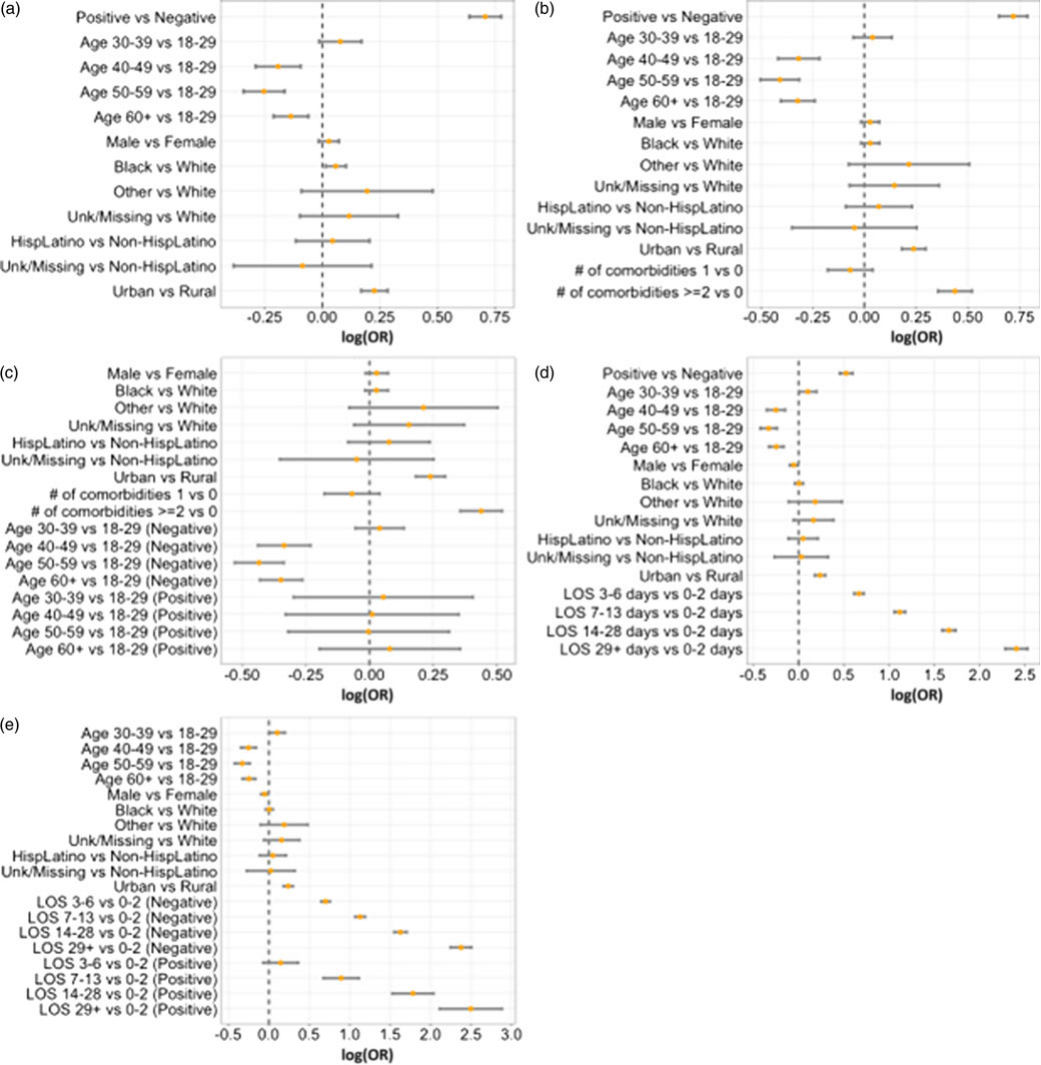
Forest plot for GLMM for all. Note: (a) Model 2 = positivity + demographics; (b) Model 3.a = Model 2+ comorbidities; (c) Model 4.a = Model 3.a+interaction between age and COVID-19 positivity; (d) Model 3.b = Model 2+ length of stay (LOS); (e) Model 4.b = Model 3.b+interaction between LOS and COVID-19 positivity.

All models demonstrated that hospitalized patients with COVID-19 diagnosis had significantly greater odds of receiving antibiotics while in the hospital (OR: 2.00 [1.87, 2.14], *p* = <0.0001, Tables 2 and 4) than those without COVID-19. In addition, patients with Black race (OR: 1.06 [1.01, 1.11], *p* = 0.0110) had greater odds of antimicrobial use in model 2 only. This association was not demonstrated after adjustments for comorbidities (OR: 1.03 [0.98, 1.08], *p* = 0.2289), and length of stay (OR: 1.01 [0.96, 1.05], *p* = 0.7735) in model 3 (Figure 2, Table S7). Residence in an urban area maintains a higher odds of antimicrobial use, whereas older age (≥60 years) had lower odds with *P* < 0.0001 throughout all the models. Number of comorbidities increases the odds of antimicrobial use (≥2 comorbidities OR: 1.55 [1.43, 1.68], *p* < 0.0001) (Model 3.a) and longer LOS increases the odds of antimicrobial use (≥29 days OR: 11.07 [9.79, 12.52], *p* < 0.0001) (Model 3.b). Male sex only showed the significant decrease in antimicrobial use when adjusting for the length of stay (*p* = 0.0299 in Model 3.b and *p* = 0.0318 in Model 4.b).

**Table 4.** Odds ratio from the GLMM models among overall population

	Model 1		Model 2		Model 3.a	
	OR	*P*-value	OR	*P*-value	OR	*P*-value
COVID status						
Negative	Ref.		Ref.		Ref.	
Positive	1.998 (1.865,2.140)	<0.0001	2.028 (1.893,2.174)	<0.0001	2.053 (1.915,2.200)	<0.0001
Age group						
18–29			Ref.		Ref.	
30–39			1.081 (0.986,1.185)	0.0976	1.039 (0.947,1.141)	0.4169
40–49			0.825 (0.748,0.910)	0.0001	0.727 (0.658,0.804)	<0.0001
50–59			0.776 (0.710,0.849)	<0.0001	0.664 (0.604,0.729)	<0.0001
≥60			0.872 (0.809,0.941)	0.0004	0.724 (0.667,0.786)	<0.0001
Gender						
Female			Ref.		Ref.	
Male			1.029 (0.984,1.075)	0.2081	1.027 (0.983,1.074)	0.2282
Race						
White			Ref.		Ref.	
Black			1.060 (1.013,1.108)	0.011	1.028 (0.983,1.075)	0.2289
Other			1.214 (0.913,1.615)	0.1826	1.239 (0.926,1.659)	0.1492
Unknown/Missing			1.123 (0.907,1.389)	0.2868	1.155 (0.930,1.434)	0.1924
Ethnicity						
Not Hispanic or Latino			Ref.		Ref.	
Hispanic or Latino			1.045 (0.891,1.227)	0.5866	1.071 (0.913,1.258)	0.3993
Unknown/Missing			0.917 (0.680,1.238)	0.5728	0.952 (0.704,1.287)	0.7474
Residence						
Rural			Ref.		Ref.	
Urban			1.253 (1.183,1.328)	<0.0001	1.268 (1.197,1.344)	<0.0001
# of comorbidity						
0					Ref.	
1					0.933 (0.837,1.040)	0.2120
≥2					1.548 (1.426,1.681)	<0.0001

	Model 4.a		Model 3.b		Model 4.b	
	OR	*P*-value	OR	*P*-value	OR	*P*-value
COVID status*						
Negative	Ref.		Ref.		Ref.	
Positive	1.438 (1.101,1.878)	0.0077	1.694 (1.577,1.819)	<0.0001	2.184 (1.809,2.637)	<0.0001
Age group**						
18–29	Ref.		Ref.		Ref.	
30–39	1.040 (0.945,1.144)	0.4225	1.109 (1.007,1.222)	0.0350	1.111 (1.009,1.224)	0.0318
40–49	0.715 (0.645,0.793)	<0.0001	0.780 (0.705,0.864)	<0.0001	0.779 (0.704,0.863)	<0.0001
50–59	0.648 (0.589,0.714)	<0.0001	0.720 (0.657,0.790)	<0.0001	0.721 (0.657,0.791)	<0.0001
≥60	0.706 (0.650,0.768)	<0.0001	0.784 (0.723,0.849)	<0.0001	0.783 (0.723,0.849)	<0.0001
Gender						
Female	Ref.		Ref.		Ref.	
Male	1.028 (0.984,1.075)	0.2153	0.951 (0.909,0.995)	0.0299	0.952 (0.910,0.996)	0.0318
Race						
White	Ref.		Ref.		Ref.	
Black	1.027 (0.982,1.075)	0.2398	1.007 (0.961,1.054)	0.7735	1.006 (0.961,1.054)	0.7929
Other	1.235 (0.923,1.652)	0.1563	1.204 (0.898,1.616)	0.2152	1.210 (0.902,1.623)	0.2043
Unknown/Missing	1.167 (0.941,1.449)	0.1598	1.181 (0.948,1.472)	0.1374	1.173 (0.942,1.462)	0.1546
Ethnicity						
Not Hispanic or Latino	Ref.		Ref.		Ref.	
Hispanic or Latino	1.079 (0.919,1.266)	0.3522	1.053 (0.895,1.239)	0.5352	1.053 (0.895,1.240)	0.5318
Unknown/Missing	0.950 (0.703,1.285)	0.7398	1.033 (0.768,1.388)	0.8314	1.026 (0.762,1.380)	0.8676
Residence						
Rural	Ref.		Ref.		Ref.	
Urban	1.269 (1.198,1.345)	<0.0001	1.270 (1.197,1.346)	<0.0001	1.272 (1.200,1.349)	<0.0001
# of comorbidity						
0	Ref.				Ref.	
1	0.933 (0.837,1.040)	0.2085	NA		0.933 (0.837,1.040)	0.2120
≥2	1.549 (1.427,1.681)	<0.0001	NA		1.548 (1.426,1.681)	<0.0001
Length of stay						
0–2			Ref.		Ref.	
3–6	NA		1.951 (1.849,2.059)	<0.0001	2.020 (1.911,2.135)	<0.0001
7–13	NA		3.058 (2.880,3.248)	<0.0001	3.093 (2.905,3.293)	<0.0001
14–28	NA		5.259 (4.878,5.670)	<0.0001	5.091 (4.703,5.512)	<0.0001
29+	NA		11.070 (9.787,12.522)	<0.0001	10.765 (9.445,12.270)	<0.0001
Interaction between age and positivity						
18–29	Ref.					
30–39	1.015 (0.707,1.456)	0.9377	NA		NA	
40–49	1.412 (0.995,2.003)	0.0531	NA		NA	
50–59	1.536 (1.110,2.124)	0.0096	NA		NA	
≥60	1.531 (1.156,2.028)	0.0030	NA		NA	
Among negatives						
Age group						
18–29	Ref.					
30–39	1.040 (0.945,1.144)	0.4225	NA		NA	
40–49	0.715 (0.645,0.793)	<0.0001	NA		NA	
50–59	0.648 (0.589,0.714)	<0.0001	NA		NA	
≥60	0.706 (0.650,0.768)	<0.0001	NA		NA	
Among positives						
Age group						
18–29	Ref.					
30–39	1.055 (0.742,1.499)	0.7656	NA		NA	
40–49	1.010 (0.719,1.418)	0.9549	NA		NA	
50–59	0.995 (0.726,1.366)	0.9775	NA		NA	
≥60	1.082 (0.821,1.426)	0.5763	NA		NA	
Interaction between LOS and positivity						
0–2					Ref.	
3–6	NA		NA		0.574 (0.459,0.717)	<0.0001
7–13	NA		NA		0.791 (0.631,0.993)	0.0433
14–28	NA		NA		1.167 (0.894,1.524)	0.2557
29+	NA		NA		1.129 (0.752,1.695)	0.5583
Among negatives						
LOS						
0–2					Ref.	
3–6	NA		NA		2.020 (1.911,2.135)	<0.0001
7–13	NA		NA		3.093 (2.905,3.293)	<0.0001
14–28	NA		NA		5.091 (4.703,5.512)	<0.0001
29+	NA		NA		10.765 (9.445,12.270)	<0.0001
Among positives						
LOS						
0–2					Ref.	
3–6	NA		NA		1.159 (0.934, 1.438)	0.1816
7–13	NA		NA		2.448 (1.968, 3.046)	<.0001
14–28	NA		NA		5.942 (4.604, 7.669)	<.0001
29+	NA		NA		12.154 (8.261, 17.880)	<.0001

The possible interaction between demographic features and COVID-19 diagnosis was reported as Model 4.a and Model 4.b, respectively. Age group has a significant interaction with COVID-19 diagnosis (Model 4.a). Older age has smaller odds of antimicrobial use among COVID negatives (≥60 years old OR: 0.71 [0.65, 0.77], *p* < 0.0001) while not significant among positives (OR: 1.08 [0.82, 1.43], *p* = 0.5763) (see Table 4). For model with LOS, the length of stay has a significant interaction with COVID-19 diagnosis (Model 4.b). Longer stay had greater odds of antimicrobial use among both positives (≥29 days OR: 12.15 [8.26, 17.89], *p* < 0.0001) and negatives (≥29 OR: 10.77 [9.45, 12.27], *p* < 0.0001) (see Table 4).

### DM2 subgroup analysis

Among those 24,551 inpatient encounters in the subgroup for DM2, there are 5,788 (23.6%) encounters with antimicrobial use (Table S1). COVID-19 diagnosis had a significant impact on antimicrobial use throughout all models. For example, COVID-19 diagnosis was associated with larger odds of antimicrobial use (OR: 2.08 [1.88, 2.31), *p* < 0.0001) in the crude model. After adjusting for demographics, number of comorbidities, and COVID-19 status (Model 3.a), older age has less odds of antimicrobial use (≥60 years old: 0.69 (0.54, 0.88); *P* = 0.0029), while COVID-19 diagnosis (OR: 2.25 [2.03, 2.49], *p* < 0.0001), male sex (OR: 1.18 [1.10, 1.26], *p* < 0.0001), urban residence (OR: 1.22 [1.12, 1.34], *p* < 0.0001), and both ≥3 comorbidities (OR: 1.64 [1.42, 1.90], *p* < 0.0001) or 2 comorbidities (OR: 1.35 [1.14, 1.59], *p* < 0.0001) had significantly higher odds of antimicrobial use (Figure S3). When adjusting LOS (Model 3.b), longer stay increased the odds of antimicrobial use (≥29 days OR: 14.10, [11.61, 17.12], *p* < 0.0001) and other covariates have similar significance to those in Model 3.a. The interaction model (Model 4.b) showed that LOS ≥29 days has greater odds of antimicrobial use among patients with COVID-19 diagnosis (OR: 12.01 [6.93, 20.80], *p* < 0.0001) while not significant odds among patients without COVID-19 diagnosis (OR: 0.87 [0.48, 1.56], *p* = 0.6405).

### Obesity subgroup analysis

Of 18,327 inpatient encounters in the subgroup for obesity, 4,771 (26.0%) had antimicrobial use (Table S4). COVID-19 diagnosis had a significant impact on antimicrobial use throughout all models. After adjusting for demographics, number of comorbidities and COVID-19 diagnosis (model 3.a), older age antimicrobial use (≥60 years old, 0.80 [0.67,0.95], *p* = 0.0095) and more comorbidities (≥3 OR: 0.86 [0.75, 0.98], *p* = 0.0282) has smaller odds of antimicrobial use while male (OR: 1.14 [1.05, 1.24], p=0.0019) and urban residence (OR: 1.31 [1.17, 1.46], *p* < 0.0001) had greater odds (Figure S4). For model with length of stay, longer stay (OR: 12.04 [9.48, 15.30], *p* < 0.0001) increased the odds of antimicrobial use significantly while male sex had no significance anymore (1.07 [0.98, 1.17], *p* = 0.1403). Other covariates have similar significance to those in Model 3.a.

## Discussion

In this administrative database cohort of SC hospitalized patients in the March 2020–April 2022 timeframe, 22% received antibiotics. This is significantly lower than the national average and may reflect robust antimicrobial stewardship practices. However, young adult patients received a significant proportion of antibiotics (24.1% in the 18–19 years old cohort, compared to ≥60 years old receiving 22.0%). Interestingly, older patients had less odds of receiving antimicrobials, at OR 0.872. When further examined interactions, the decrease is also noted in the COVID-19-negative older patients. This may represent the inclusion of a significant number of old patients admitted for noninfectious causes (i.e. cerebrovascular events, heart failure exacerbations, and renal failure) who received COVID-19 testing due to admission screening requirements. In turn, this may suggest that younger patients are more likely to be admitted for an infectious-related diagnosis, while older patients are admitted to hospital for a wide variety of illnesses. Numbers of hospitalizations for young people (18–64 years) decreased by 18.4% in April–June 2020, but in-hospital deaths increased by 36.4% in the same time period; continued increases were seen with 27.3% increase in July-September and 45.5% increase in October–December 2020.^10^ This population-based data indicated the severity of illness of those being admitted to the hospital, and suggests likely cognitive or motivational biases driving antimicrobial prescribing in “sick” patients.^11^

COVID-19 diagnosis influenced receipt of antimicrobials; 34.9% of patients with COVID-19 diagnosis received antimicrobials compared to 21.4% of those without COVID-19 diagnosis (*p* < 0.0001). There is significant misuse of antimicrobials in hospitalized patients with viral infections. There is significantly high use of broad-spectrum, hospital-onset antibiotics with vancomycin and cefepime being extremely high use in frequency compared to the COVID-negative cohort (Figure 1). This trend of antimicrobials used for hospital-onset infections was described early in the pandemic in SC previously; it is concerning that it continued through 2022.^6^ Also alarming is the high use of azithromycin compared to the COVID-negative cohort, as well as a smaller though meaningful difference in doxycycline between the cohorts (Figure 1). Each individual healthcare system provided guidelines for treatment of COVID-19 patients in SC, particularly early in the pandemic, though generally, they were in line with the National Institutes of Health guidelines due to rapid changes. Throughout the pandemic, there have been significant concerns about empiric antimicrobial prescribing that may have been due to the diversion of stewardship efforts away from core activities during the pandemic.^12^ Also note that aggressive stewardship efforts have occurred around patients with COVID-19, with 82% of respondents in a survey reporting monitoring antimicrobial use in COVID-19.^13^ Baseline stewardship activities are critical, not just in monitoring in COVID-19 patients but in all patients. Ongoing work is necessary to continue to guard against inappropriate and unnecessary antimicrobial use in viral infections, though obviously secondary or superinfections would require treatment.

These models were an attempt to also better understand disparities in prescribing antimicrobials that may have been exacerbated during the last few years. There is a dearth of data regarding racial or ethnic differences in antimicrobial use to further explore why those categorized as Black race received more antibiotics in this inpatient cohort, though there are well-established reports of structural racism affecting social determinants of health and population health in the United States.^14,15^ Black patients are at higher risk for having obesity, diabetes, hypertension, and cardiovascular disease than Whites; they also have less access to primary care and have historically received inadequate care even with similar conditions and insurance as White patients.^16,17^ Additionally, racial and ethnic disparities in healthcare access have been widely seen during the COVID-19 pandemic. The quality of medication prescribing and access to appropriate medications, including stewardship for appropriate antimicrobial use, is a critical aspect of achieving pharmacoequity across all races and ethnicities.^17^ Notably, race and ethnicity are limited as to how the EMR and intake healthcare workers collect this data and must be further explored to better understand how different social groups experience medical care including antimicrobials. This may explain the differences seen in the different models with those categorized as Black—in some models, it is a significant variable, while not in others; namely an overall higher odds of antimicrobial use in the category of Black race, which was not demonstrated after adjustments for comorbidities (OR: 1.03 [0.98, 1.08], *p* = 0.2289) and length of stay (OR: 1.01 [0.96, 1.05], *p* = 0.7735) in model 3 (Figure 2, Table S7).

Additional disparities may exist in residence, generally categorized as urban or rural. There are noted disparities in health for rural residents, exacerbated by the pandemic including crumbling hospital infrastructures with diminished access to ICU beds, infectious diseases specialists, and specialized care.^18^ Rural patients are traditionally shown to be prescribed more antibiotics for common conditions.^19,20^ The Healthcare Cost and Utilization Project describes the overall number of hospitalization and in-hospital deaths for patients in rural areas decreased by 19.4% in the April-June 2020 (second quarter) time frame, when compared to historical data; deaths increased by 14.3% and 71.4% in the third and fourth quarters of 2020, respectively.^21^ Urban residents increased the odds of receipt of antimicrobials by 25% in this cohort; this relationship disappeared when adjusting for COVID-19 status and comorbidities. This may reflect the urban nature of this healthcare system data, and different relationships may be seen in other geographic locations. This also could be an ecologic fallacy seen in this particular cohort but not on an individual level and is worth additional analyses.

Frailer patients, as one could generalize those with more comorbid conditions, have significantly higher utilization of antibiotics—1.5 odds of receiving antibiotics if ≥2 comorbid conditions. Both DM2 and obesity subgroup analyses had 22 or 26% utilization of antibiotics, respectively; these subgroups also showed higher odds of antimicrobial use in male patients than female patients, which bears further exploration. Regardless of gender, the number of comorbid conditions increasing antimicrobial use is consistent with other studies from early in COVID-19, March-June 2020, showing patients more likely to have received antibacterial therapy if they were older, had lower body mass index, more severe illness, lobar infiltrate, or were admitted to a for-profit hospital.^22^ Additionally, an Italian survey shows that there is significant variation in prescriber practices depending on their own prescribing habits, and ASP principles remain at risk of being overlooked due to personal preferences.^23^ This is worth exploring further, as this may be limited by the binary nature of receipt of antimicrobials without the clinical context of short-term empiric use compared to ongoing treatment use.

Length of stay significantly impacted antimicrobial use but is unclear if this is confounding, in light of the known increase in healthcare onset infections during 2020 and 2021^24,25^. We attempted to categorize the LOS to capture common durations of antimicrobial prescriptions. If patients were in hospital >29 days, their odds of receiving antimicrobials were 11.07. Longer LOS, critical care stay, and receipt of mechanical ventilation contribute to incidence of hospital-onset antimicrobial-resistant infections among inpatients with COVID-19.^25^ Longer LOS not only increases odds for healthcare-associated infection but a hospital-onset antimicrobial-resistant one and therefore is a reminder about the safety of healthcare in the United States and the threat of antimicrobial resistance.

There are significant limitations in this data. First, there is a concern in using administrative data, regarding the accuracy of the data, and regarding appropriate input to ensure the final data is accurate. Antimicrobial use is more dynamic than just receipt or non-receipt, but the use of claims data in this only allows for more binary outcomes. This is an overall limitation to the utilization of machine learning in administrative data sets in infectious diseases. There was also no ability to extract microbiologic data to help assess appropriateness of antimicrobial prescribing. This study also was only inclusive of one major medical system in SC and these data may not be extrapolated or applicable to other states or regions. While we theoretically had access to antimicrobial start/stop data, there were noted inaccuracies in the dataset on closer examination. Additionally, due to the concern for being able to identify patients, the exact date of health care visit was shifted by 1–365 days. Therefore, no inferences can be made about timing during the COVID-19 pandemic e.g. different “waves” or “surges.”

As such, ecologic or population studies such as this are subject to “ecological correlation,” which may be fallacies (ecologic fallacy) in misattributing population-level behaviors when there are differences on an individual level.^26^ This may explain why some variables changed in significance in the different models as we attempted to drill down on specific group characteristics. Antimicrobial use is incredibly complex, and additional studies with more individual-level data should be undertaken to better understand which populations receive antimicrobials.

There are significant relationships around antimicrobial prescribing that need to be reassessed and addressed since the COVID-19 pandemic has changed medical practices, along with more healthcare-associated infections and ongoing concerns about worsening antimicrobial resistance. A renewed focus on antimicrobial stewardship principles to ensure antimicrobial use is appropriate is critical, while addressing both population-based needs and more individual practices.

## Supporting information

Bailey et al. supplementary material Bailey et al. supplementary material

## Supplementary material

For supplementary material accompanying this paper visit http://doi.org/10.1017/ash.2023.496. click here to view supplementary material
