# Supplementary material for: Ecologic analysis of antimicrobial use in South Carolina hospitals during 2020–2022
Source: Antimicrob Steward Healthc Epidemiol. 2023 Dec 12;3(1):e232. doi: 10.1017/ash.2023.496 (PMC10753502; doi:10.1017/ash.2023.496)
Supplement: Bailey et al. supplementary material [file S2732494X23004965sup001.docx]

Supplement:

Figure S1: Top 10 frequency of antimicrobial prescription by COVID-19 status for subset DM2

Figure S2: Top 10 frequency of antimicrobial prescription by COVID-19 status for subset obesity

Figure S3: Forest plot for GLMM for DM subset


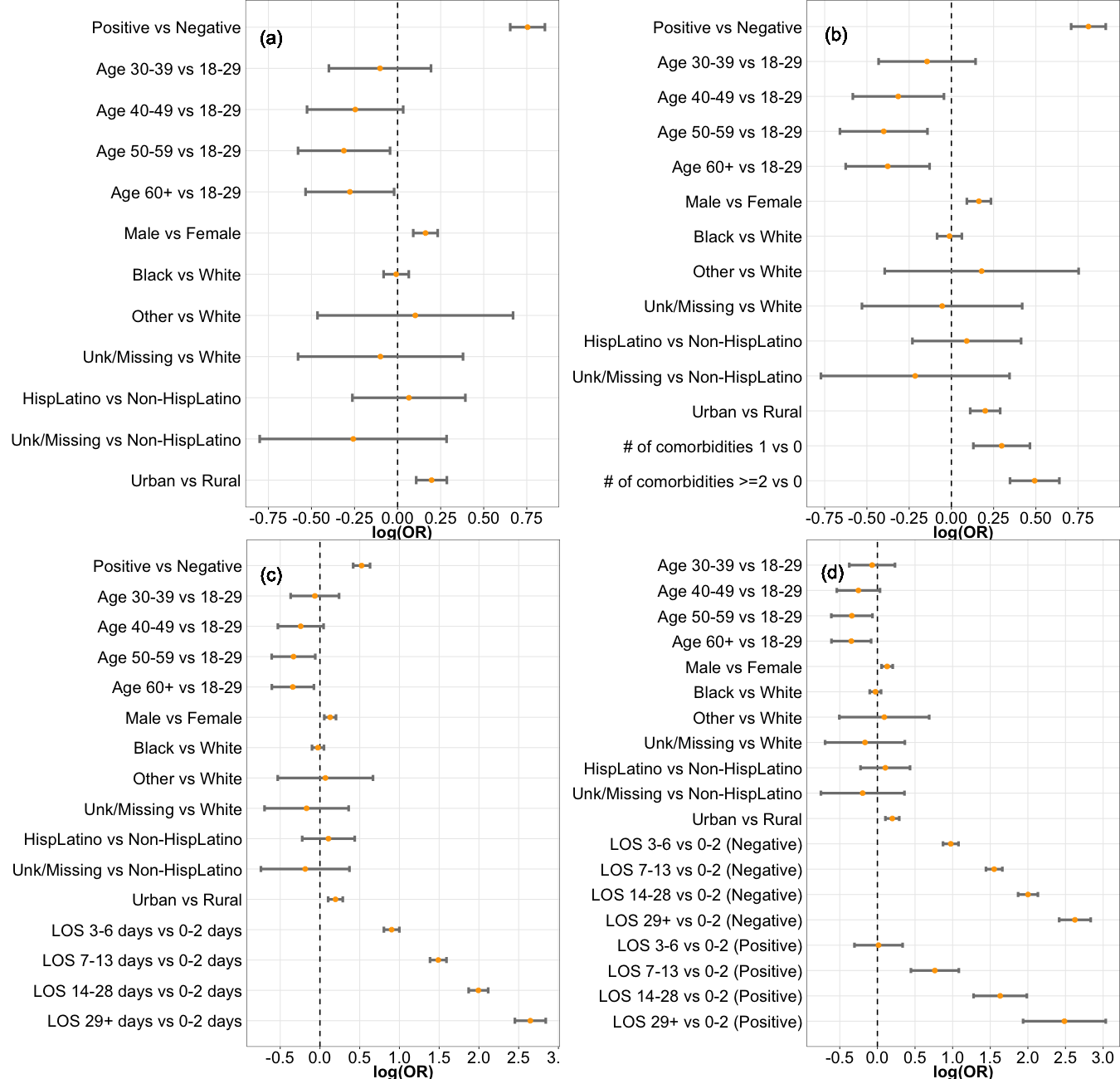


Note: (a) Model 2 = positivity + demographics; (b) Model 3.a = Model 2+# of comorbidities; (c) Model 3.b = Model 2+length of stay (LOS); (d) Model 4.b = Model 3.b+interaction between LOS and Covid-19 positivity.

Figure S4: Forest plot for GLMM for Obesity subset


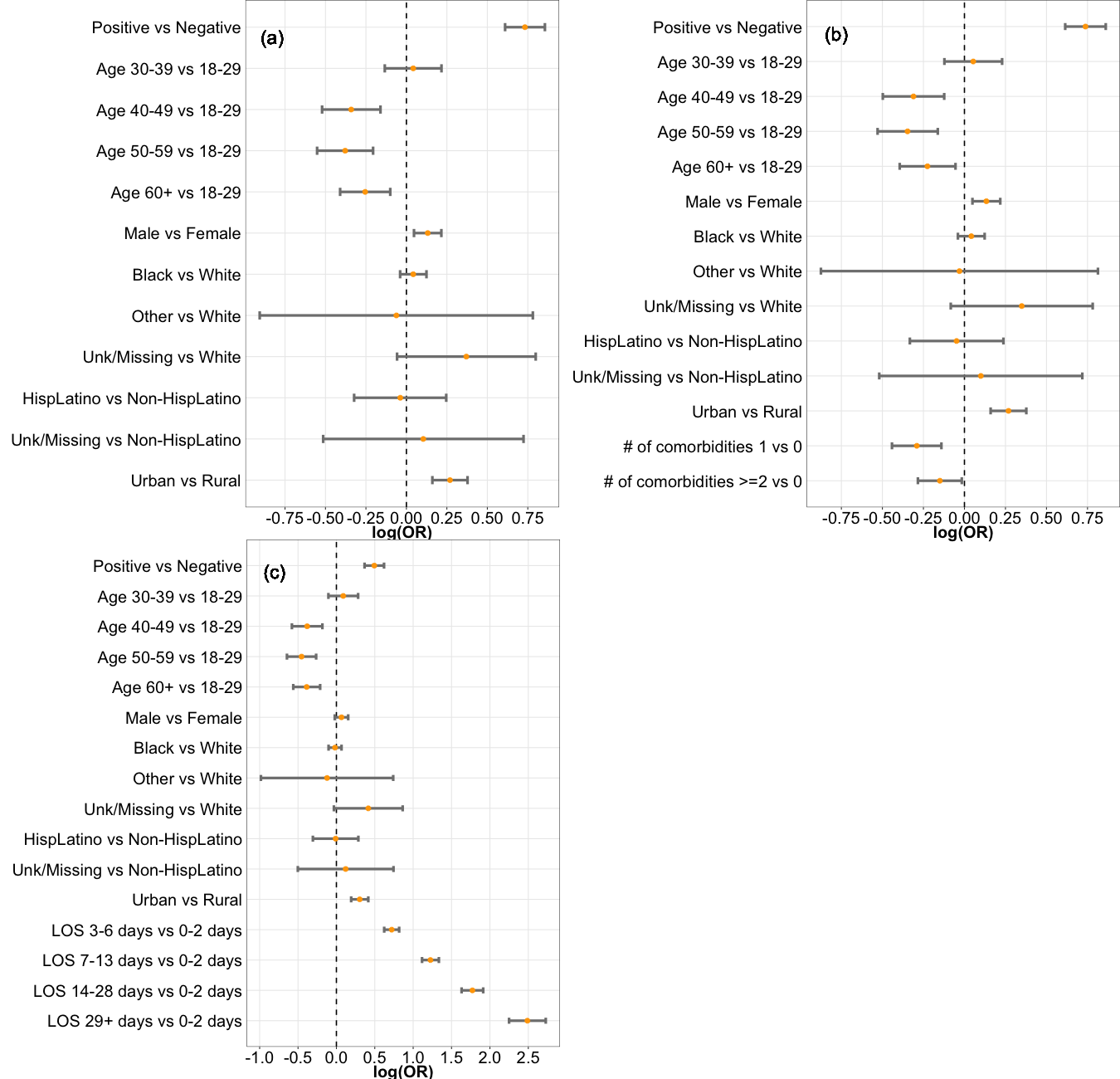


Note: (a) Model 2 = positivity + demographics; (b) Model 3.a = Model 2+# of comorbidities; (c) Model 3.b = Model 2+length of stay (LOS).

Table S1: Demographic characteristics of the overall inpatient encounters among the population with DM2

| **Characteristics** | **Overall** | **No med** | **Any med** | **p-value** |
| --- | --- | --- | --- | --- |
|  | 24,551 | 18,763 | 5,788 |  |
|  |  | 76.42% | 23.58% |  |
| Age group |  |  |  | 0.0086 |
| 18-29 | 682 (2.78) | 493 (2.63) | 189 (3.27) |  |
| 30-39 | 1,496 (6.09) | 1,109 (5.91) | 387 (6.69) |  |
| 40-49 | 2,551 (10.39) | 1,952 (10.40) | 599 (10.35) |  |
| 50-59 | 4,565 (18.59) | 3,536 (18.85) | 1,029 (17.78) |  |
| >=60 | 15,257 (62.14) | 11,673 (62.21) | 3,584 (61.92) |  |
| Gender |  |  |  | <.0001 |
| Female | 12,845 (52.32) | 9,972 (53.15) | 2,873 (49.64) |  |
| Male | 11,706 (47.68) | 8,791 (46.85) | 2,915 (50.36) |  |
| Race |  |  |  | 0.9077 |
| White | 11,532 (46.97) | 8,809 (46.95) | 2,723 (47.05) |  |
| Black | 12,747 (51.92) | 9,751 (51.97) | 2,996 (51.76) |  |
| Other | 81 (0.33) | 60 (0.32) | 21 (0.36) |  |
| Unknown/Missing | 191 (0.78) | 143 (0.76) | 48 (0.83) |  |
| Ethnicity |  |  |  | 0.236 |
| Not Hispanic or Latino | 24,037 (97.91) | 18,375 (97.93) | 5,662 (97.82) |  |
| Hispanic or Latino | 411 (1.67) | 304 (1.62) | 107 (1.85) |  |
| Unknown/Missing | 103 (0.42) | 84 (0.45) | 19 (0.33) |  |
| Residence |  |  |  | <.0001 |
| Rural | 5,687 (23.16) | 4,492 (23.94) | 1,195 (20.65) |  |
| Urban | 18,864 (76.84) | 14,271 (76.06) | 4,593 (79.35) |  |
| Covid-19 status |  |  |  | <.0001 |
| Negative | 22,641 (92.22) | 17,565 (93.62) | 5,076 (87.70) |  |
| Positive | 1,910 (7.78) | 1,198 (6.38) | 712 (12.30) |  |
| # of comorbidities |  |  |  | <.0001 |
| 1 | 1,685 (6.86) | 1,377 (7.34) | 308 (5.32) |  |
| 2 | 3,181 (12.96) | 2,474 (13.19) | 707 (12.21) |  |
| >=3 | 19,685 (80.18) | 14,912 (79.48) | 4,773 (82.46) |  |
| Length of stay (days) |  |  |  | <.0001 |
| 0-2 | 6,464 (26.33) | 5,841 (31.13) | 623 (10.76) |  |
| 3-6 | 10,251 (41.75) | 8,076 (43.04) | 2,175 (37.58) |  |
| 7-13 | 5,138 (20.93) | 3,463 (18.46) | 1,675 (28.94) |  |
| 14-28 | 2,118 (8.63) | 1,160 (6.18) | 958 (16.55) |  |
| 29+ | 580 (2.36) | 223 (1.19) | 357 (6.17) |  |

Table S2: Demographic characteristics of the Covid-19 positive encounters among population with DM2

| **Characteristics** | **Overall** | **No med** | **Any med** | **p-value** |
| --- | --- | --- | --- | --- |
|  | 1,910 | 1,198 | 712 |  |
|  |  | 62.72% | 37.28% |  |
| Age group |  |  |  | 0.7908 |
| 18-29 | 27 (1.41) | 14 (1.17) | 13 (1.83) |  |
| 30-39 | 67 (3.51) | 44 (3.67) | 23 (3.23) |  |
| 40-49 | 159 (8.32) | 101 (8.43) | 58 (8.15) |  |
| 50-59 | 291 (15.24) | 184 (15.36) | 107 (15.03) |  |
| >=60 | 1,366 (71.52) | 855 (71.37) | 511 (71.77) |  |
| Gender |  |  |  | 0.0013 |
| Female | 1,022 (53.51) | 675 (56.34) | 347 (48.74) |  |
| Male | 888 (46.49) | 523 (43.66) | 365 (51.26) |  |
| Race |  |  |  | 0.0017** |
| White | 692 (36.23) | 472 (39.40) | 220 (30.90) |  |
| Black | 1,176 (61.57) | 700 (58.43) | 476 (66.85) |  |
| Other | * | * | * |  |
| Unknown/Missing | * | * | * |  |
| Ethnicity |  |  |  | 0.8938 |
| Not Hispanic or Latino | 1,849 (96.81) | 1,158 (96.66) | 691 (97.05) |  |
| Hispanic or Latino | * | * | * |  |
| Unknown/Missing | * | * | * |  |
| Residence |  |  |  | 0.0224 |
| Rural | 519 (27.17) | 347 (28.96) | 172 (24.16) |  |
| Urban | 1,391 (72.83) | 851 (71.04) | 540 (75.84) |  |
| # of comorbidity |  |  |  | 0.0016 |
| 1 | 271 (14.19) | 195 (16.28) | 76 (10.67) |  |
| 2 | 396 (20.73) | 252 (21.04) | 144 (20.22) |  |
| >=3 | 1,243 (65.08) | 751 (62.69) | 492 (69.10) |  |
| Length of stay (days) |  |  |  | <.0001 |
| 0-2 | 272 (14.24) | 207 (17.28) | 65 (9.13) |  |
| 3-6 | 710 (37.17) | 536 (44.74) | 174 (24.44) |  |
| 7-13 | 535 (28.01) | 321 (26.79) | 214 (30.06) |  |
| 14-28 | 296 (15.50) | 114 (9.52) | 182 (25.56) |  |
| 29+ | 97 (5.08) | 20 (1.67) | 77 (10.81) |  |

Note: * Small number less than 10 were masked due to DHEC’s policy.

** p-value from Fisher’s exact test

Table S3: Demographic characteristics of the COVID-19 negative encounters among population with DM2

| **Characteristics** | **Overall** | **No med** | **Any med** | **p-value** |
| --- | --- | --- | --- | --- |
|  | 22,641 | 17,565 | 5,076 |  |
|  |  | 77.58% | 22.42% |  |
| Age group |  |  |  | 0.0015 |
| 18-29 | 655 (2.89) | 479 (2.73) | 176 (3.47) |  |
| 30-39 | 1,429 (6.31) | 1,065 (6.06) | 364 (7.17) |  |
| 40-49 | 2,392 (10.56) | 1,851 (10.54) | 541 (10.66) |  |
| 50-59 | 4,274 (18.88) | 3,352 (19.08) | 922 (18.16) |  |
| >=60 | 13,891 (61.35) | 10,818 (61.59) | 3,073 (60.54) |  |
| Gender |  |  |  | <.0001 |
| Female | 11,823 (52.22) | 9,297 (52.93) | 2,526 (49.76) |  |
| Male | 10,818 (47.78) | 8,268 (47.07) | 2,550 (50.24) |  |
| Race |  |  |  | 0.1104 |
| White | 10,840 (47.88) | 8,337 (47.46) | 2,503 (49.31) |  |
| Black | 11,571 (51.11) | 9,051 (51.53) | 2,520 (49.65) |  |
| Other | 74 (0.33) | 55 (0.31) | 19 (0.37) |  |
| Unknown/Missing | 156 (0.69) | 122 (0.69) | 34 (0.67) |  |
| Ethnicity |  |  |  | 0.2543 |
| Not Hispanic or Latino | 22,188 (98.00) | 17,217 (98.02) | 4,971 (97.93) |  |
| Hispanic or Latino | 359 (1.59) | 270 (1.54) | 89 (1.75) |  |
| Unknown/Missing | 94 (0.42) | 78 (0.44) | 16 (0.32) |  |
| Residence |  |  |  | <.0001 |
| Rural | 5,168 (22.83) | 4,145 (23.60) | 1,023 (20.15) |  |
| Urban | 17,473 (77.17) | 13,420 (76.40) | 4,053 (79.85) |  |
| # of comorbidity |  |  |  | <.0001 |
| 1 | 1,414 (6.25) | 1,182 (6.73) | 232 (4.57) |  |
| 2 | 2,785 (12.30) | 2,222 (12.65) | 563 (11.09) |  |
| >=3 | 18,442 (81.45) | 14,161 (80.62) | 4,281 (84.34) |  |
| Length of stay (days) |  |  |  | <.0001 |
| 0-2 | 6,192 (27.35) | 5,634 (32.08) | 558 (10.99) |  |
| 3-6 | 9,541 (42.14) | 7,540 (42.93) | 2,001 (39.42) |  |
| 7-13 | 4,603 (20.33) | 3,142 (17.89) | 1,461 (28.78) |  |
| 14-28 | 1,822 (8.05) | 1,046 (5.96) | 776 (15.29) |  |
| 29+ | 483 (2.13) | 203 (1.16) | 280 (5.52) |  |

Table S4: Demographic characteristics of the overall encounters among population with obesity

| **Characteristics** | **Overall** | **No med** | **Any med** | **p-value** |
| --- | --- | --- | --- | --- |
|  | 18,327 | 13,556 | 4,771 |  |
|  |  | 73.97% | 26.03% |  |
| Age group |  |  |  | <.0001 |
| 18-29 | 1,495 (8.16) | 1,045 (7.71) | 450 (9.43) |  |
| 30-39 | 2,207 (12.04) | 1,519 (11.21) | 688 (14.42) |  |
| 40-49 | 2,618 (14.28) | 1,990 (14.68) | 628 (13.16) |  |
| 50-59 | 3,584 (19.56) | 2,742 (20.23) | 842 (17.65) |  |
| >=60 | 8,423 (45.96) | 6,260 (46.18) | 2,163 (45.34) |  |
| Gender |  |  |  | 0.0297 |
| Female | 11,392 (62.16) | 8,489 (62.62) | 2,903 (60.85) |  |
| Male | 6,935 (37.84) | 5,067 (37.38) | 1,868 (39.15) |  |
| Race |  |  |  | 0.0117 |
| White | 9,200 (50.20) | 6,869 (50.67) | 2,331 (48.86) |  |
| Black | 8,941 (48.79) | 6,564 (48.42) | 2,377 (49.82) |  |
| Other | * | * | * |  |
| Unknown/Missing | * | * | * |  |
| Ethnicity |  |  |  | 0.3209 |
| Not Hispanic or Latino | 17,885 (97.59) | 13,242 (97.68) | 4,643 (97.32) |  |
| Hispanic or Latino | 374 (2.04) | 264 (1.95) | 110 (2.31) |  |
| Unknown/Missing | 68 (0.37) | 50 (0.37) | 18 (0.38) |  |
| Residence |  |  |  | <.0001 |
| Rural | 3,804 (20.76) | 2,967 (21.89) | 837 (17.54) |  |
| Urban | 14,523 (79.24) | 10,589 (78.11) | 3,934 (82.46) |  |
| Covid-19 status |  |  |  | <.0001 |
| Negative | 17,133 (93.49) | 12,846 (94.76) | 4,287 (89.86) |  |
| Positive | 1,194 (6.51) | 710 (5.24) | 484 (10.14) |  |
| # of comorbidities |  |  |  | <.0001 |
| 1 | 1,652 (9.01) | 1,123 (8.28) | 529 (11.09) |  |
| 2 | 2,390 (13.04) | 1,801 (13.29) | 589 (12.35) |  |
| >=3 | 14,285 (77.95) | 10,632 (78.43) | 3,653 (76.57) |  |
| Length of stay (days) |  |  |  | <.0001 |
| 0-2 | 5,245 (28.62) | 4,523 (33.37) | 722 (15.13) |  |
| 3-6 | 7,797 (42.54) | 5,865 (43.26) | 1,932 (40.49) |  |
| 7-13 | 3,578 (19.52) | 2,347 (17.31) | 1,231 (25.80) |  |
| 14-28 | 1,329 (7.25) | 691 (5.10) | 638 (13.37) |  |
| 29+ | 378 (2.06) | 130 (0.96) | 248 (5.20) |  |

Note: * Small number less than 10 were masked due to DHEC’s policy.

Table S5: Demographic characteristics of the COVID-19 positive encounters among population with obesity

| **Characteristics** | **Overall** | **No med** | **Any med** | **p-value** |
| --- | --- | --- | --- | --- |
|  | 1,194 | 710 | 484 |  |
|  |  | 59.46% | 40.54% |  |
| Age group |  |  |  | 0.2687 |
| 18-29 | 79 (6.62) | 46 (6.48) | 33 (6.82) |  |
| 30-39 | 111 (9.30) | 68 (9.58) | 43 (8.88) |  |
| 40-49 | 150 (12.56) | 93 (13.10) | 57 (11.78) |  |
| 50-59 | 240 (20.10) | 155 (21.83) | 85 (17.56) |  |
| >=60 | 614 (51.42) | 348 (49.01) | 266 (54.96) |  |
| Gender |  |  |  | 0.0024 |
| Female | 764 (63.99) | 479 (67.46) | 285 (58.88) |  |
| Male | 430 (36.01) | 231 (32.54) | 199 (41.12) |  |
| Race |  |  |  | 0.0960* |
| White | 430 (36.01) | 273 (38.45) | 157 (32.44) |  |
| Black | 745 (62.40) | 427 (60.14) | 318 (65.70) |  |
| Other | * | * | * |  |
| Unknown/Missing | * | * | * |  |
| Ethnicity |  |  |  | 0.3166** |
| Not Hispanic or Latino | 1,154 (96.65) | 683 (96.20) | 471 (97.31) |  |
| Hispanic or Latino | * | * | * |  |
| Unknown/Missing | * | * | * |  |
| Residence |  |  |  | 0.0371 |
| Rural | 289 (24.20) | 187 (26.34) | 102 (21.07) |  |
| Urban | 905 (75.80) | 523 (73.66) | 382 (78.93) |  |
| # of comorbidity |  |  |  | 0.0117 |
| 1 | 127 (10.64) | 85 (11.97) | 42 (8.68) |  |
| 2 | 218 (18.26) | 143 (20.14) | 75 (15.50) |  |
| >=3 | 849 (71.11) | 482 (67.89) | 367 (75.83) |  |
| Length of stay (days) |  |  |  | <.0001 |
| 0-2 | 153 (12.81) | 118 (16.62) | 35 (7.23) |  |
| 3-6 | 486 (40.70) | 347 (48.87) | 139 (28.72) |  |
| 7-13 | 319 (26.72) | 183 (25.77) | 136 (28.10) |  |
| 14-28 | 175 (14.66) | * | * |  |
| 29+ | 61 (5.11) | * | * |  |

Note: * Small number less than 10 were masked due to DHEC’s policy.

** p-value from Fisher’s exact test

Table S6: Demographic characteristics of the COVID-19 negative encounters among population with obesity

| **Characteristics** | **Overall** | **No med** | **Any med** | **p-value** |
| --- | --- | --- | --- | --- |
|  | 17,133 | 12,846 | 4,287 |  |
|  |  | 74.98% | 25.02% |  |
| Age group |  |  |  | <.0001 |
| 18-29 | 1,416 (8.26) | 999 (7.78) | 417 (9.73) |  |
| 30-39 | 2,096 (12.23) | 1,451 (11.30) | 645 (15.05) |  |
| 40-49 | 2,468 (14.40) | 1,897 (14.77) | 571 (13.32) |  |
| 50-59 | 3,344 (19.52) | 2,587 (20.14) | 757 (17.66) |  |
| >=60 | 7,809 (45.58) | 5,912 (46.02) | 1,897 (44.25) |  |
| Gender |  |  |  | 0.1331 |
| Female | 10,628 (62.03) | 8,010 (62.35) | 2,618 (61.07) |  |
| Male | 6,505 (37.97) | 4,836 (37.65) | 1,669 (38.93) |  |
| Race |  |  |  | 0.1237 |
| White | 8,770 (51.19) | 6,596 (51.35) | 2,174 (50.71) |  |
| Black | 8,196 (47.84) | 6,137 (47.77) | 2,059 (48.03) |  |
| Other | 27 (0.16) | * | * |  |
| Unknown/Missing | 140 (0.82) | * | * |  |
| Ethnicity |  |  |  | 0.2399 |
| Not Hispanic or Latino | 16,731 (97.65) | 12,559 (97.77) | 4,172 (97.32) |  |
| Hispanic or Latino | 337 (1.97) | 240 (1.87) | 97 (2.26) |  |
| Unknown/Missing | 65 (0.38) | 47 (0.37) | 18 (0.42) |  |
| Residence |  |  |  | <.0001 |
| Rural | 3,515 (20.52) | 2,780 (21.64) | 735 (17.14) |  |
| Urban | 13,618 (79.48) | 10,066 (78.36) | 3,552 (82.86) |  |
| # of comorbidity |  |  |  | <.0001 |
| 1 | 1,525 (8.90) | 1,038 (8.08) | 487 (11.36) |  |
| 2 | 2,172 (12.68) | 1,658 (12.91) | 514 (11.99) |  |
| >=3 | 13,436 (78.42) | 10,150 (79.01) | 3,286 (76.65) |  |
| Length of stay (days) |  |  |  | <.0001 |
| 0-2 | 5,092 (29.72) | 4,405 (34.29) | 687 (16.03) |  |
| 3-6 | 7,311 (42.67) | 5,518 (42.96) | 1,793 (41.82) |  |
| 7-13 | 3,259 (19.02) | 2,164 (16.85) | 1,095 (25.54) |  |
| 14-28 | 1,154 (6.74) | 635 (4.94) | 519 (12.11) |  |
| 29+ | 317 (1.85) | 124 (0.97) | 193 (4.50) |  |

Note: * Small numbers less than 10 were masked due to DHEC’s policy.

Table S7: Odds ratio from the GLMM models among DM2 patients

|  | Model 1 | | Model 2 | | Model 3.a | |
| --- | --- | --- | --- | --- | --- | --- |
|  | OR | P-value | OR | P-value | OR | P-value |
| Covid status |  |  |  |  |  |  |
| Negative | Ref. |  | Ref. |  | Ref. |  |
| Positive | 2.084 (1.884,2.305) | <0.0001 | 2.127 (1.923,2.352) | <0.0001 | 2.251 (2.032,2.494) | <0.0001 |
| Age group |  |  |  |  |  |  |
| 18-29 |  |  | Ref. |  | Ref. |  |
| 30-39 |  |  | 0.903 (0.671,1.214) | 0.4986 | 0.866 (0.650,1.154) | 0.327 |
| 40-49 |  |  | 0.782 (0.591,1.033) | 0.0832 | 0.730 (0.558,0.957) | 0.0226 |
| 50-59 |  |  | 0.732 (0.561,0.957) | 0.0223 | 0.670 (0.517,0.868) | 0.0024 |
| >=60 |  |  | 0.758 (0.586,0.980) | 0.0347 | 0.686 (0.535,0.879) | 0.0029 |
| Gender |  |  |  |  |  |  |
| Female |  |  | Ref. |  | Ref. |  |
| Male |  |  | 1.176 (1.095,1.262) | <0.0001 | 1.177 (1.096,1.264) | <0.0001 |
| Race |  |  |  |  |  |  |
| White |  |  | Ref. |  | Ref. |  |
| Black |  |  | 0.992 (0.922,1.067) | 0.8212 | 0.989 (0.919,1.064) | 0.7659 |
| Other |  |  | 1.108 (0.628,1.955) | 0.7238 | 1.196 (0.674,2.124) | 0.54 |
| Unknown/Missing |  |  | 0.905 (0.561,1.462) | 0.6838 | 0.947 (0.589,1.521) | 0.8212 |
| Ethnicity |  |  |  |  |  |  |
| Not Hispanic or Latino |  |  | Ref. |  | Ref. |  |
| Hispanic or Latino |  |  | 1.068 (0.769,1.482) | 0.6954 | 1.095 (0.794,1.511) | 0.58 |
| Unknown/Missing |  |  | 0.773 (0.449,1.329) | 0.3514 | 0.807 (0.462,1.411) | 0.4525 |
| Residence |  |  |  |  |  |  |
| Rural |  |  | Ref. |  | Ref. |  |
| Urban |  |  | 1.218 (1.114,1.331) | <0.0001 | 1.222 (1.118,1.335) | <0.0001 |
| # of comorbidity |  |  |  |  |  |  |
| 1 |  |  |  |  | Ref. |  |
| 2 |  |  |  |  | 1.347 (1.139,1.592) | 0.0005 |
| >=3 |  |  |  |  | 1.637 (1.415,1.895) | <0.0001 |

Table S7 (continue): Odds ratio from the GLMM models among DM2 patients

|  | Model 3.b | | Model 4.b | |
| --- | --- | --- | --- | --- |
|  | OR | P-value | OR | P-value |
| Covid status |  |  |  |  |
| Negative | Ref. |  | Ref. |  |
| Positive | 1.689 (1.519,1.878) | <0.0001 | 3.290 (2.471,4.379) | <0.0001 |
| Age group |  |  |  |  |
| 18-29 |  |  | Ref. |  |
| 30-39 | 0.938 (0.692,1.272) | 0.6808 | 0.932 (0.688,1.263) | 0.6513 |
| 40-49 | 0.785 (0.589,1.047) | 0.1 | 0.777 (0.583,1.035) | 0.085 |
| 50-59 | 0.717 (0.545,0.943) | 0.0173 | 0.712 (0.542,0.936) | 0.0149 |
| >=60 | 0.711 (0.546,0.927) | 0.0116 | 0.707 (0.543,0.920) | 0.0098 |
| Gender |  |  |  |  |
| Female |  |  | Ref. |  |
| Male | 1.137 (1.056,1.224) | 0.0006 | 1.138 (1.057,1.226) | 0.0006 |
| Race |  |  |  |  |
| White |  |  | Ref. |  |
| Black | 0.975 (0.905,1.052) | 0.5186 | 0.974 (0.903,1.050) | 0.4961 |
| Other | 1.071 (0.588,1.949) | 0.823 | 1.096 (0.603,1.991) | 0.7635 |
| Unknown/Missing | 0.845 (0.498,1.435) | 0.5336 | 0.848 (0.499,1.440) | 0.5415 |
| Ethnicity |  |  |  |  |
| Not Hispanic or Latino |  |  | Ref. |  |
| Hispanic or Latino | 1.113 (0.800,1.549) | 0.5261 | 1.111 (0.799,1.543) | 0.5314 |
| Unknown/Missing | 0.831 (0.476,1.450) | 0.515 | 0.822 (0.472,1.433) | 0.4897 |
| Residence |  |  |  |  |
| Rural |  |  | Ref. |  |
| Urban | 1.216 (1.109,1.333) | <0.0001 | 1.219 (1.112,1.337) | <0.0001 |
| LOS |  |  |  |  |
| 0-2 |  |  |  |  |
| 3-6 | 2.464 (2.235,2.716) | <0.0001 | 2.651 (2.392,2.939) | <0.0001 |
| 7-13 | 4.430 (3.994,4.913) | <0.0001 | 4.720 (4.233,5.264) | <0.0001 |
| 14-28 | 7.340 (6.492,8.300) | <0.0001 | 7.401 (6.490,8.441) | <0.0001 |
| 29+ | 14.102 (11.614,17.123) | <0.0001 | 13.810 (11.200,17.029) | <0.0001 |
| Interaction between LOS and positivity |  |  |  |  |
| 0-2 |  |  |  |  |
| 3-6 | NA |  | 0.383 (0.273,0.536) | <0.0001 |
| 7-13 | NA |  | 0.455 (0.325,0.636) | <0.0001 |
| 14-28 | NA |  | 0.691 (0.474,1.007) | 0.0545 |
| 29+ | NA |  | 0.870 (0.484,1.563) | 0.6405 |
| Among negatives |  |  |  |  |
| 0-2 |  |  |  |  |
| 3-6 | NA |  | 0.383 (0.273,0.536) | <0.0001 |
| 7-13 | NA |  | 0.455 (0.325,0.636) | <0.0001 |
| 14-28 | NA |  | 0.691 (0.474,1.007) | 0.0545 |
| 29+ | NA |  | 0.870 (0.484,1.563) | 0.6405 |
| Among positives |  |  |  |  |
| 0-2 |  |  |  |  |
| 3-6 | NA |  | 1.015 (0.737, 1.398) | 0.9267 |
| 7-13 | NA |  | 2.146 (1.561, 2.951) | <.0001 |
| 14-28 | NA |  | 5.115 (3.590, 7.286) | <.0001 |
| 29+ | NA |  | 12.010 (6.934, 20.802) | <.0001 |

Table S8: Odds ratio from the GLMM models among obesity patients

|  | Model 1 | |
| --- | --- | --- |
|  | OR | P-value |
| Covid status |  |  |
| Negative | Ref. |  |
| Positive | 2.037 (1.802,2.302) | <0.0001 |

Table S8 (continue): Odds ratio from the GLMM models among obesity patients

|  | Model 2 | | Model 3.a | | Model 3.b | |
| --- | --- | --- | --- | --- | --- | --- |
|  | OR | P-value | OR | P-value | OR | P-value |
| Covid status |  |  |  |  |  |  |
| Negative | Ref. |  | Ref. |  | Ref. |  |
| Positive | 2.080 (1.840,2.353) | <0.0001 | 2.091 (1.848,2.366) | <0.0001 | 1.638 (1.443,1.860) | <0.0001 |
| Age group |  |  |  |  |  |  |
| 18-29 |  |  | Ref. |  | Ref. |  |
| 30-39 | 1.043 (0.875,1.242) | 0.6389 | 1.055 (0.885,1.258) | 0.5493 | 1.093 (0.901,1.327) | 0.3661 |
| 40-49 | 0.711 (0.594,0.852) | 0.0002 | 0.733 (0.608,0.884) | 0.0011 | 0.683 (0.560,0.833) | 0.0002 |
| 50-59 | 0.685 (0.576,0.814) | <0.0001 | 0.707 (0.589,0.850) | 0.0002 | 0.635 (0.525,0.768) | <0.0001 |
| >=60 | 0.775 (0.664,0.905) | 0.0012 | 0.798 (0.674,0.947) | 0.0095 | 0.679 (0.570,0.809) | <0.0001 |
| Gender |  |  |  |  |  |  |
| Female |  |  | Ref. |  | Ref. |  |
| Male | 1.141 (1.048,1.241) | 0.0023 | 1.143 (1.050,1.244) | 0.0019 | 1.068 (0.979,1.166) | 0.1403 |
| Race |  |  |  |  |  |  |
| White |  |  | Ref. |  | Ref. |  |
| Black | 1.043 (0.962,1.132) | 0.3061 | 1.043 (0.961,1.131) | 0.3144 | 0.982 (0.903,1.069) | 0.6801 |
| Other | 0.940 (0.404,2.184) | 0.885 | 0.970 (0.417,2.256) | 0.9433 | 0.885 (0.374,2.096) | 0.782 |
| Unknown/Missing | 1.448 (0.944,2.223) | 0.0902 | 1.418 (0.920,2.185) | 0.1131 | 1.515 (0.968,2.371) | 0.0694 |
| Ethnicity |  |  |  |  |  |  |
| Not Hispanic or Latino |  |  | Ref. |  | Ref. |  |
| Hispanic or Latino | 0.963 (0.724,1.279) | 0.7933 | 0.953 (0.717,1.268) | 0.743 | 0.990 (0.736,1.331) | 0.9461 |
| Unknown/Missing | 1.110 (0.598,2.062) | 0.7414 | 1.105 (0.595,2.051) | 0.7528 | 1.128 (0.605,2.104) | 0.7039 |
| Residence |  |  |  |  |  |  |
| Rural |  |  | Ref. |  | Ref. |  |
| Urban | 1.309 (1.174,1.459) | <0.0001 | 1.308 (1.173,1.458) | <0.0001 | 1.355 (1.212,1.515) | <0.0001 |
| # of comorbidity |  |  |  |  |  |  |
| 1 |  |  | Ref. |  |  |  |
| 2 |  |  | 0.748 (0.643,0.869) | 0.0002 | NA |  |
| >=3 |  |  | 0.861 (0.753,0.984) | 0.0282 | NA |  |
| LOS |  |  |  |  |  |  |
| 0-2 |  |  |  |  |  |  |
| 3-6 |  |  |  |  | 2.055 (1.864,2.265) | <0.0001 |
| 7-13 |  |  |  |  | 3.405 (3.052,3.800) | <0.0001 |
| 14-28 |  |  |  |  | 5.881 (5.109,6.770) | <0.0001 |
| 29+ |  |  |  |  | 12.044 (9.484,15.295) | <0.0001 |

Table S9: ICD-10 code for comorbidities.

| Comorbidities | ICD-10 |
| --- | --- |
| Myocardial infarction | I21.x, I22.x, I25.2 |
| Congestive heart failure | I09.9, I11.0, I13.0, I13.2, I25.5, I42.x, I43.x, I50.x, P29.0 |
| Peripheral vascular disease | I70.x, I71.x, I73.1, I73.8, I73.9, I77.1, I79.0, I79.1, I79.8, K55.1, K55.8, K55.9, Z95.8, Z95.9 |
| Cerebrovascular disease | G45.x, G46.x, H34.0, H34.1, H34.2, I60.x, I61.x, I62.x, I63.x, I64.x, I65.x, I66.x, I67.x, I68.x |
| Dementia | F01.x, F02.x, F03.x, F04.x, F05.x, F06.1, F06.8, G13.2, G13.8, G30.x, G31.0, G31.1, G31.2, G91.4, G94.x, R41.81, R54.x |
| Chronic pulmonary disease | J40.x, J41.x, J42.x, J43.x, J44.x, J45.x, J46.x, J47.x, J60.x, J61.x, J62.x, J63.x, J64.x, J65.x, J66.x, J67.x, J68.4, J70.1, J70.3 |
| Rheumatic disease | M05.x, M06.x, M31.5, M32.x, M33.x, M34.x, M35.1, M35.3, M36.0 |
| Peptic ulcer disease | K25.x, K26.x, K27.x |
| Mild liver disease | B18.x, K70.0, K70.1, K70.2, K70.3, K70.9, K71.3, K71.4, K71.5, K71.7, K73.x, K74.x, K76.0, K76.2, K76.3, K76.4, K76.8, K76.9, Z94.4 |
| Moderate or severe liver disease | I85.0, I86.4, K70.4, K71.1, K72.1, K72.9, K76.5, K76.6, K76.7 |
| Diabetes | E08.x, E09.x, E10.x, E11.x, E13.x, Ex.0x, Ex.1x, Ex.2x, Ex.3x, Ex.4x, Ex.5x, Ex.6x, Ex.8x, Ex.9x |
| Renal (mild or moderate) | I12.9, I13.0, I13.10, N03.x, N05.x, N18.1, N18.2, N18.3, N18.4, N18.9, Z94.0 |
| Renal (severe) | I12.0, I13.11, I13.2, N18.5, N18.6, N19.x, N25.0, Z49.x, Z99.2 |
| Hemiplegia or paraplegia | G04.1, G11.4, G80.0, G80.1, G80.2, G81.x, G82.x, G83.x |
| Cancer | C0x.x, C1x.x, C2x.x, C30.x, C31.x, C32.x, C33.x, C34.x, C37.x, C38.x, C39.x, C40.x, C41.x, C43.x, C45.x, C46.x, C47.x, C48.x, C49.x, C50.x, C51.x, C52.x, C53.x, C54.x, C55.x, C56.x, C57.x, C58.x, C60.x, C61.x, C62.x, C63.x, C76.x, C80.1C81.x, C82.x, C83.x, C84.x, C85.x, C88.x, C9x.x |
| Metastatic solid tumor | C77.x, C78.x, C79.x, C80.0, C80.2 |
| HIV/AIDS | B20, B21, B22, B24 |
| Obesity | E66.x |
